# Effects of ginkgo leaf tablet on the pharmacokinetics of rosiglitazone in rats and its potential mechanism

**DOI:** 10.1080/13880209.2022.2087688

**Published:** 2022-06-25

**Authors:** Xueting Xing, Mengzhu Kong, Qiaoyu Hou, Jiaqi Li, Wen Qian, Xijing Chen, Hanhan Li, Changqing Yang

**Affiliations:** a School of Basic Medicine and Clinical Pharmacy, China Pharmaceutical University, Nanjing, China; b Department of Pharmacy, Nanjing Drum Tower Hospital, The Affiliated Hospital of Nanjing University Medical School, Jiangsu, China; c Nanjing BRT-Biomed Company, Limited, Jiangning District, Jiangsu Province, China

**Keywords:** CYP2C8, CYP2C9, drug-drug interaction, metabolism

## Abstract

**Context:**

Ginkgo leaf tablet (GLT), a traditional Chinese herbal formula, is often combined with rosiglitazone (ROS) for type 2 diabetes mellitus treatment. However, the drug-drug interaction between GLT and ROS remains unknown.

**Objective:**

To investigate the effects of GLT on the pharmacokinetics of ROS and its potential mechanism.

**Materials and methods:**

The pharmacokinetics of 10 mg/kg ROS with 100/200 mg/kg GLT as single-dose and 10-day multiple-dose administration were investigated in Sprague-Dawley rats. *In vitro*, the effects of GLT on the activity of CYP2C8 and CYP2C9 were determined in recombinant human yeast microsomes and rat liver microsomes with probe substrates.

**Results:**

The *t*_1/2_ of ROS increased from 2.14 ± 0.38 (control) to 2.79 ± 0.37 (100 mg/kg) and 3.26 ± 1.08 h (200 mg/kg) in the single-dose GLT administration. The AUC_0-t_ (139.69 ± 45.46 vs. 84.58 ± 39.87 vs. 66.60 ± 15.90 h·μg/mL) and *t*_1/2_ (2.75 ± 0.70 vs. 1.99 ± 0.44 vs. 1.68 ± 0.35 h) decreased significantly after multiple-dose GLT treatment. The IC_50_ values of quercetin, kaempferol, and isorhamnetin, GLT main constituents, were 9.32, 7.67, and 11.90 μmol/L for CYP2C8, and 27.31, 7.57, and 4.59 μmol/L for CYP2C9. The multiple-dose GLT increased rat CYP2C8 activity by 44% and 88%, respectively.

**Discussion and conclusions:**

The metabolism of ROS is attenuated in the single dose of GLT by inhibiting CYP2C8 and CYP2C9 activity, and accelerated after the multiple-dose GLT treatment via inducing CYP2C8 activity in rats, indicating that the clinical dose of ROS should be adjusted when co-administrated with GLT.

## Introduction

Rosiglitazone (ROS), a thiazolidinedione insulin sensitiser, could be used for the treatment of type 2 diabetes mellitus due to the enhanced sensitivity of peripheral tissue to insulin and improved utilisation of glucose through activating peroxisome proliferator‑activated receptor γ (Dawed et al. 2016; Matsumoto et al. 2019; Ren et al. 2020; Zhou et al. 2021). It is rapidly absorbed, almost completely bioavailable from the gastrointestinal tract (Cox et al. 2000). ROS is metabolised mainly by CYP2C8 via *N*-demethylation and *p*-hydroxylation and by CYP2C9 to a lesser extent in the human liver (Naik et al. 2012; Wring et al. 2018). The metabolites are considerably less potent than the parent form (Park et al. 2004). Therefore, modulation of CYP2C8 or CYP2C9 activities may play a vital role in the pharmacokinetic profiles of ROS.

Ginkgo leaf tablet (GLT) is one of the most widely sold and studied medicinal plant preparations in the world (Rao et al. 2014). The active component in GLT is *Ginkgo biloba* L. (Ginkgoaceae) extract, which mainly consists of 22–27% flavone glycosides (the primary active ingredients including glycosides of quercetin, kaempferol, and isorhamnetin) and 5–7% terpene lactones (ginkgolides and bilobalide) (Ude et al. 2013; Guan et al. 2014). GLT could be used as a potential therapy for a variety of diseases, such as diabetic cardiomyopathy, neurodegenerative diseases, myocardial lesion, cancer, obesity, and liver injury (Hirata et al. 2019; Martinez-Solis et al. 2019; Achete de Souza et al. 2020). The intake of GLT may induce or inhibit hepatic drug metabolising enzymes and consequently alter the metabolism, clearance, and response of co-administered drugs. The effects of GLT on the activities of cytochrome P450 (CYP) subtype enzymes (CYP1A1, CYP1A2, CYP2B, CYP2C9, CYP2C19, CYP2E1, and CYP3A4) have been investigated in previous research (Sugiyama et al. 2004; Tang et al. 2007; Deng et al. 2016; Wang et al. 2016). For example, the combination of GLT and amlodipine could cause the reduction of amlodipine clearance rate and the increase of amlodipine plasma concentration, implying that GLT influences the metabolism of amlodipine by inhibiting the activity of CYP3A4 (Wang et al. 2016). However, the co-administration of GLT and theophylline could reduce the plasma concentration of theophylline and weaken its efficacy (Tang et al. 2007). In clinical practice, GLT is used in combination with various drugs in high frequency, which could result in drug-drug interaction. However, the effects of GLT on the pharmacokinetics of ROS remain unknown.

The present study evaluates the effects of GLT on the pharmacokinetics of ROS after oral administration of GLT as a single dose or multiple doses and uses probe assay methods in recombinant human CYP2C8 (RHCYP2C8) and CYP2C9 (RHCYP2C9) yeast microsomes, and rat liver microsomes (RLM) to further explore the main potential mechanism.

## Materials and methods

### Reagents

ROS (purity > 99%) was purchased from the Macklin Biochemical Co., Ltd. (Shanghai, China). GLT was provided by Yangtze River Pharmaceutical Group Co., Ltd. (Taizhou, Jiangsu, China). Diclofenac (purity > 98%) was purchased from the Tokyo Chemical Industry (Tokyo, Japan). Amodiaquine, quercetin, kaempferol, and isorhamnetin were supplied by Wuhan Yuan Cheng Technology Co., Ltd. (Wuhan, Hubei, China). The internal standard testosterone (purity > 98%) and carbamazepine (purity > 97%) were obtained from Sinopharm Chemical Reagent Co., Ltd. (Shanghai, China) and Beijing Bailingwei Technology Co., Ltd. (Beijing, China), respectively. Both RHCYP2C8 and RHCYP2C9 yeasts were purchased from Nanjing BRT-Biomed Co., Ltd. (Nanjing, Jiangsu, China). The NADPH regeneration system was purchased from iPhase Biosciences Co., Ltd. (Beijing, China). Lowry Protein Assay Kit was obtained from Beijing Solarbio Science & Technology Co., Ltd. (Beijing, China). Acetonitrile was obtained from Merck Drugs & Biotechnology (chromatographic grade; Woodbridge, NJ, USA). Ultrapure water was prepared by the Milli-Q water purification system (Millipore, Billerica, MA, USA). All other chemicals were of analytical grade or better.

### *In vivo* study

#### Animals

All the experimental procedures involving animals were approved by the Ethics Committee of the China Pharmaceutical University (Nanjing, Jiangsu, China) (No.2021-09-020). Male Sprague-Dawley rats weighing 180–200 g were supplied by Zhejiang Experimental Animal Centre (Hangzhou, Zhejiang, China). The rats were housed five per cage and provided with food and water *ad libitum* in air-conditioned animal quarters at 22 ± 2 °C and 50 ± 10% relative humidity with a 12 h light/dark cycle. The animals were acclimatised to the facilities for 5 d and then fasted with free access to water for 12 h prior to each experiment.

#### A single-dose or multiple-dose GLT with ROS in rats

For the pharmacokinetic study *in vivo*, 36 rats were equally randomised to 6 groups, including control group (ROS 10 mg/kg + vehicle) group (A, D), ROS (10 mg/kg) + GLT (100 mg/kg) group (B, E), and ROS (10 mg/kg) + GLT (200 mg/kg) group (C, F). The doses of GLT and ROS were determined by combining the references and the medicinal product specification (Muzeeb et al. 2006; Wang et al. 2016). The ROS and GLT fraction powders were all homogenised in the vehicle (0.5% CMC-Na solution containing 1% Tween 80). For the single dose, Group A, B, and C were treated with vehicle (5 mL/kg), normal-dose GLT suspension (100 mg/kg), and high-dose GLT suspension (200 mg/kg) by oral gavage, respectively. Thirty-min later, ROS was orally administered to rats in each group. For multiple doses, Group D, E, and F were pre-treated with a vehicle, normal-dose GLT suspension, and high-dose GLT suspension, respectively, by oral gavage for 10 consecutive days. On the 11th day, half an hour after GLT administration, ROS was orally administered to rats in each group. The blood samples (0.25 mL) were collected in heparin tubes via the oculi chorioideae vein at 0, 0.25, 0.5, 0.75, 1, 1.5, 2, 3, 4, 6, 8 10, 12, and 24 h after ROS administration.

After centrifuging at 12,000 rpm for 5 min, the plasma samples (100 μL) were then spiked with 10 μL of the testosterone (100 μg/mL) and extracted with 500 μL of ethyl acetate by vortexing for 3 min. The mixer was centrifuged at 12,000 rpm for 10 min and then the superior organic phase was transferred to a conic plastic centrifugal tube and dried under nitrogen at 30 °C. The residue was dissolved in 50 μL methanol for HPLC analysis.

### *In vitro* study

#### Preparation of RHCYP2C8 and RHCYP2C9 microsomes

RHCYP2C8 and RHCYP2C9 yeast microsomes were extracted using the glass bead crushing method and differential centrifugation, with grinding 15 times by 0.5 g glass beads and centrifugation at 9,000 *g* for 20 min at 4 °C. The supernatant was then centrifuged at 100,000 *g* at 4 °C (BECKMAN COULTER Optima™ L-80 XP Ultracentrifuge) for 1 h, and the precipitation was RHCYP2C8 or RHCYP2C9 yeast microsome. Reconstituted with potassium phosphate buffer (pH 7.4), the microsome solution was collected and stored at −80 °C for subsequent examination. Protein concentrations of microsomes were determined using the Lowry method.

#### Preparation of RLM

Fifteen rats were randomised into 3 groups (*n* = 5 per group), receiving vehicle or GLT at 100 or 200 mg/kg daily by gavage for 10 consecutive days. On day 11, rats were sacrificed, and livers were harvested. The livers were weighed, and 2 volumes of ice-cold homogenisation buffer (50 mmol/L Tris-HCl buffer at pH 7.4 containing 0.25 mmol/L sucrose and 1 mmol/L EDTA) were then added. The homogenate was then centrifuged at 16,000 *g* for 20 min. The supernatants were then transferred to a new tube and centrifuged at 100,000 *g* for 1 h. The microsomal pellets were resuspended with Tris-HCl buffer containing 20% glycerol and stored at −80 °C until use. Protein concentrations of microsomes were determined using the Lowry method.

#### Microsomal metabolism of amodiaquine and diclofenac

Amodiaquine and diclofenac are typical CYP2C8 and CYP2C9 probe substrates, respectively. The inhibitory effects of quercetin, kaempferol, and isorhamnetin on the activity of RHCYP2C8 and RHCYP2C9 were determined. The 0.4 mL incubation mixtures contained inhibitors (0–500 μmol/L), potassium phosphate buffer (pH 7.4) in common, and RHCYP2C8 (12.4 mg/mL) with amodiaquine or RHCYP2C9 (4.6 mg/mL) with diclofenac. Table 1 showed the concentration range of the inhibitors. The reactions were initiated by the addition of NADPH with pre-incubation 10 min in advance and carried out for 40 min.

**Table 1.** The concentration range of quercetin, kaempferol, and isorhamnetin in the IC_50_ study for RHCYP2C8 and RHCYP2C9.

P450 enzyme	Substrate	Inhibitors	
		Name	Tested concentration (μmol/L)
RHCYP2C8	Amodiaquine	Quercetin	0.5, 1, 2.5, 5, 10, 25, 50, 100
		Kaempferol	0.1, 0.5, 1, 2.5, 5, 10, 25, 50, 100, 500
		Isorhamnetin	0.1, 0.5, 1, 2.5, 5, 10, 25, 50, 100, 500
RHCYP2C9	Diclofenac	Quercetin	2.5, 5, 10, 25, 50, 100, 200, 500, 1000
		Kaempferol	0.1, 0.5, 2.5, 5, 10, 25, 50, 100, 400
		Isorhamnetin	0.025, 0.1, 0.5, 1, 2.5, 10, 25, 50, 100, 400

The activities of CYP2C8 and CYP2C9 were investigated in liver microsomes from rats treated with GLT at 100 or 200 mg/kg. The 0.4 mL incubation mixtures contained microsomal protein (2.3 mg/mL), 10 μmol/L amodiaquine or diclofenac, and potassium phosphate buffer (pH 7.4). After 10-min-pre-incubation at 37 °C, incubation was initiated by the addition of NADPH and carried out for 40 min.

The CYP2C8 sample treatment process was that incubation was terminated by adding 200 μL of methanol. Samples were centrifuged at 12,000 rpm for 10 min, and the supernatant was subjected to HPLC. The CYP2C9 sample treatment process was that each sample (100 μL) was spiked with 10 μL of carbamazepine (30 μg/mL). The mixture was then extracted with 500 μL ethyl acetate by vortexing for 3 min and centrifuged at 12,000 rpm for 10 min. The superior organic phase was transferred to a conic plastic centrifuge tube and dried under nitrogen at 30 °C. The residue was dissolved in 100 μL methanol for HPLC analysis.

### Chromatographic conditions

Samples were analysed on a SHIMAZU LC-2010AHT HPLC system equipped with an ultraviolet absorption detector, and the separations were performed on a Galaksil® EF-C18H (4.6 mm × 250 mm, 5 μm) column for ROS or Galaksil® EF-C18H (4.6 mm × 150 mm, 5 μm) column for desethyl amodiaquine (DEAQ) or Inertsil® ODS-SP (4.6 mm × 150 mm, 5 μm) column for 4-hydroxy diclofenac (4-OH DCF) at 30 °C. The mobile phase was composed of acetonitrile:0.02 mol/L ammonium acetate (50:50, v/v, adjusted pH to 6.0 with acetic acid) for ROS or methanol:0.1 mol/L ammonium acetate (9:91, v/v, adjusted pH to 3.0 with acetic acid) for DEAQ or 0.1%TFA water: 0.1%TFA acetonitrile (49:51, v/v) for 4-OH DCF with the flow rate at 1.0 mL/min. The detection wavelength of samples for ROS, DEAQ, and 4-OH DCF was 247, 340, and 280 nm and with injection volumes at 20, 10, and 10 μL.

### Validation of HPLC method

#### Specificity

Specificity of the established method was investigated by analysing blank plasma samples from six individual rats, which were compared to those obtained by the spiking ROS and testosterone into the corresponding blank plasma sample and those collected from rat plasma after 1 h of drug administration to monitor interference.

#### Linearity and limit of quantification (LOQ)

For the calibration curve, ten concentrations of calibration standards (0.025, 0.05, 0.1, 0.5, 1, 2.5, 5, 10, 25, and 50 μg/mL) were processed as described above. The calibration curves for ROS were established by plotting peak area ratios of the ROS to testosterone against plasma concentrations. LOQ was determined as the concentration of the ROS with a signal-to-noise ratio of 10.

#### Precision and accuracy

To determine intra-day precision and accuracy, nine replicates of quality control (QC) samples at three different concentration levels (low, 0.025 μg/mL; medium, 25 μg/mL; high, 50 μg/mL) were analysed on the same day. Inter-day precision was evaluated on three independent days. The intra- and inter-day precisions were represented by the relative standard deviation (RSD) value.

### Statistical analysis

All raw data were analysed on the Shimadzu LabSolution software. The pharmacokinetic parameters were calculated using the Phoenix WinNonlin 6.4 software (Certara Inc., Princeton, NJ, USA). The data in this study were presented as mean ± SD for the individual groups. An unpaired Student’s *t*-test was used to determine any significant differences. A *p*-value less than 0.05 was considered statistically significant (*p < 0.05; **p < 0.01). Statistical analysis and IC_50_ values were conducted and calculated using GraphPad Prism version 7.0 (San Diego, CA, USA).

## Results

### Method validation

#### Selectivity

**Figure 1.**
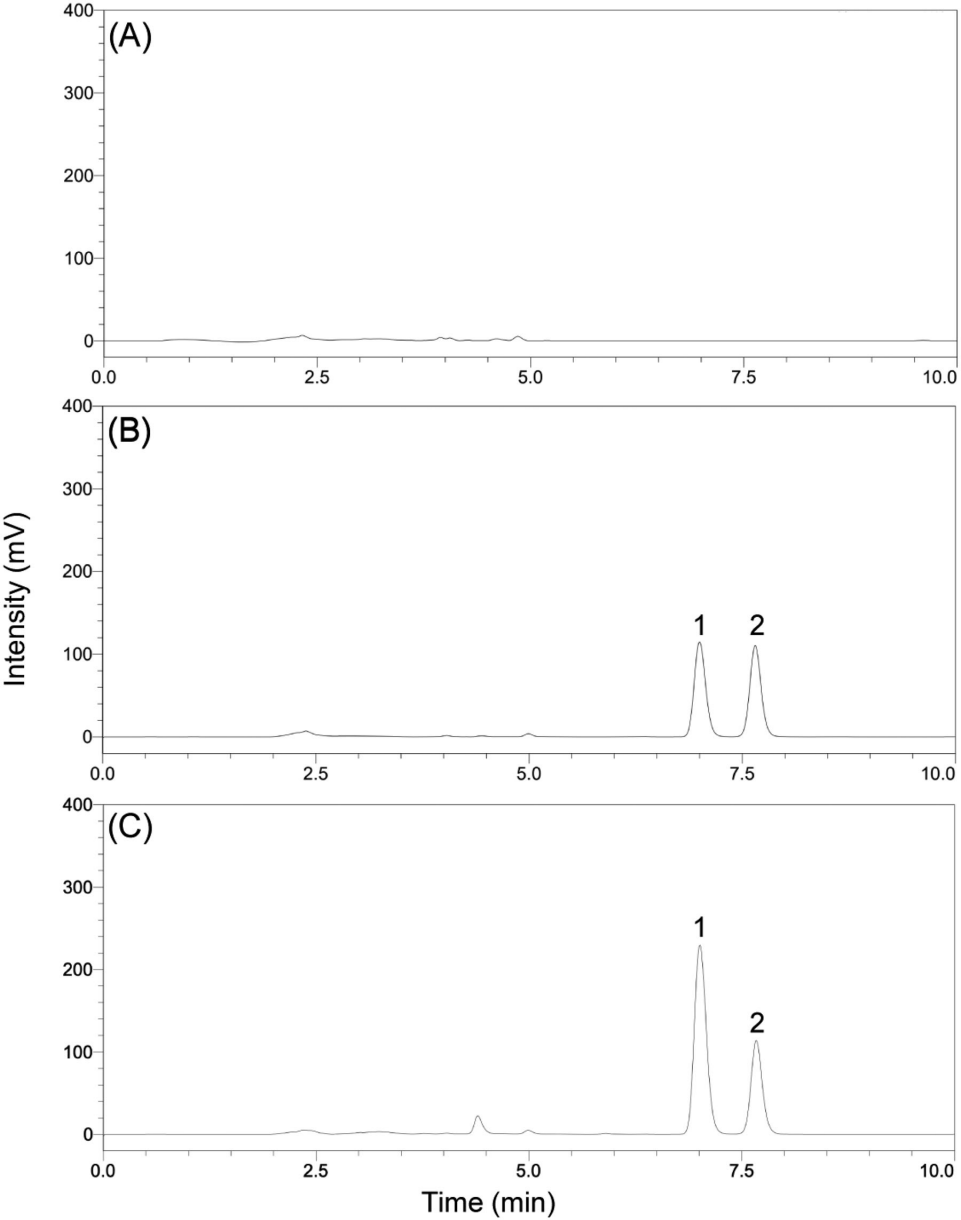
(A) Representative chromatogram of blank plasma; (B) The chromatograms obtained from plasma spiked with 10 μg/mL for ROS (1) and 10 μg/mL for testosterone (2); (C) The chromatograms obtained from rat plasma 1 h after drug administration.

In the present study, the selectivity was examined by making use of independent plasma samples from six different rats. As shown in Figure 1, no obvious interference was observed in the representative chromatogram of a blank plasma sample at the retention times of the ROS and testosterone.

#### Linearity and LOQ

Linearity for ROS was obtained over the concentration range of 0.025–50 μg/mL. A typical calibration curve was *y* = 0.1077x + 0.0002 (*r*^2^ = 0.998), where y represents the peak area ratios of ROS to the testosterone and x represents the plasma concentrations of ROS. The LOQ of ROS was detected to be 0.025 μg/mL in rat plasma.

#### Precision and accuracy

In this study, QC samples at low, medium, and high concentration levels (0.025, 25, and 50 μg/mL) were prepared to determine the precision and accuracy. The intra-day precision was 3.85%, 4.85%, and 1.47%, while the inter-day precision was 3.20%, 3.73%, and 4.27%, respectively. There was accuracy of 104.00 ± 4.00% in low concentration levels, 101.77 ± 4.94% in medium concentration levels, and 100.15 ± 1.47% for high concentration levels (Table 2). The intra- and inter-day precision values (RSD) were below 10%. These data indicated that the method was satisfactory with stability and reliability.

**Table 2.** The intra-day and inter-day and accuracy of ROS in plasma samples (*n* = 3).

ROS concentration (μg/mL)	Intra-day precision (%)	Inter-day precision (%)	Accuracy (%)
0.025	3.85	3.20	104.00 ± 4.00
25	4.85	3.73	101.77 ± 4.94
50	1.47	4.27	100.15 ± 1.47

### Effect of a single-dose GLT on the pharmacokinetics of ROS in rats

**Figure 2.**
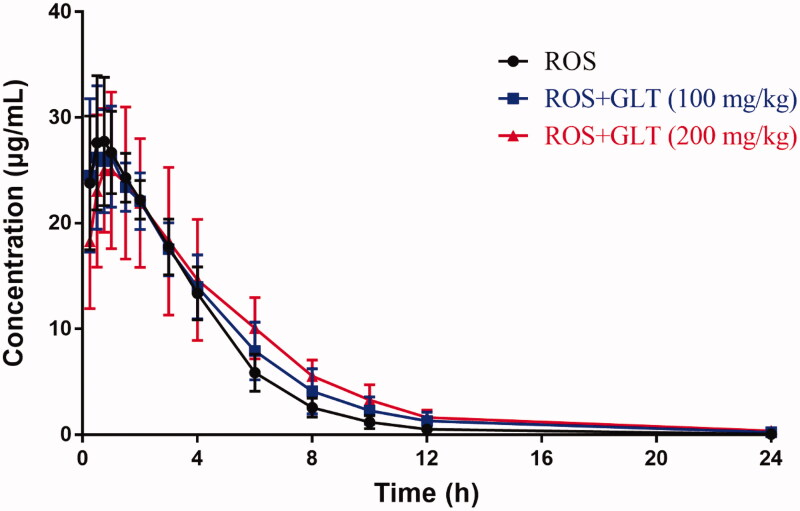
Mean plasma ROS (10 mg/kg) concentration-time curves with a single-dose vehicle or GLT administration in rats (*n* = 6).

The mean plasma ROS concentration-time curves treated with a single-dose vehicle or GLT (100 or 200 mg/kg) are shown in Figure 2, and the pharmacokinetic parameters of ROS in rats are shown in Table 3. After the rats were treated with GLT (100 or 200 mg/kg), the *t*_1/2_ increased from 2.14 ± 0.38 to 2.79 ± 0.37 and 3.26 ± 1.08 h with an increase of 30% and 52% (*p* < 0.05). In addition, the MRT significantly expanded from 3.02 ± 0.28 to 3.66 ± 0.63 and 4.24 ± 1.04 h, with the extension of 21% and 40% (*p* < 0.05), respectively. The results indicated that the *t*_1/2_ and MRT of ROS were dose-dependent. However, there were no significant differences in AUC_0-t_, *C*_max_, and *T*_max_ of ROS.

**Table 3.** Pharmacokinetic parameters of ROS (10 mg/kg) in the control group and single-dose GLT groups (*n* = 6).

Parameters	Control	GLT (100 mg/kg)	GLT (200 mg/kg)
*t*_1/2_ (h)	2.14 ± 0.38	2.79 ± 0.37*	3.26 ± 1.08*
CL/F (mL/h)	16.46 ± 1.91	14.58 ± 2.12	13.97 ± 3.43
MRT (h)	3.02 ± 0.28	3.66 ± 0.63*	4.24 ± 1.04*
AUC_0-t_ (h·μg/mL)	116.64 ± 12.18	129.66 ± 24.28	139.17 ± 40.64
*C*_max_ (μg/mL)	29.12 ± 5.98	28.82 ± 5.86	26.05 ± 6.99
*T*_max_ (h)	0.90 ± 0.34	1.01 ± 0.65	0.80 ± 0.26

*p < 0.05, **p < 0.01, compared with control.

### Effect of multiple-dose GLT on the pharmacokinetics of ROS in rats

**Figure 3.**
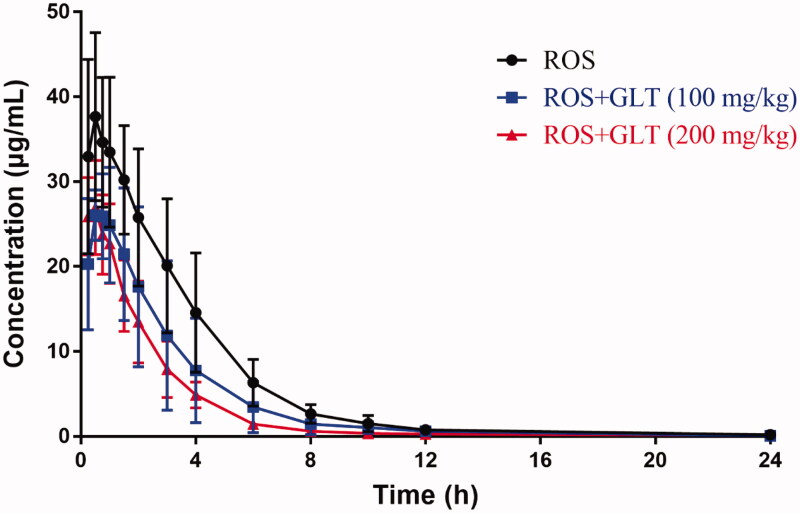
Mean ROS (10 mg/kg) concentration-time curves in rat plasma after GLT multiple-dose administration (*n* = 6).

The mean plasma ROS concentration-time curves pre-treated with vehicle and GLT (100 or 200 mg/kg) for 10 days are shown in Figure 3, and the pharmacokinetic parameters of ROS in rats are shown in Table 4. Compared with the control group, the GLT treatment groups (100 and 200 mg/kg) were significantly decreased in *C*_max_ (from 38.72 ± 9.73 to 28.51 ± 5.56 and 28.41 ± 4.64 μg/mL) with a reduction of 26% and 27%, and AUC_0-t_ (from 139.69 ± 45.46 to 84.58 ± 39.87 and 66.60 ± 15.90 h·μg/mL) with a reduction of 39% and 52% (*p* < 0.05). Besides, a significant increase in CL/F was observed as evidenced by the value from 13.92 ± 3.04 to 25.54 ± 9.02 and 30.00 ± 8.52 mL/h. Moreover, the *t*_1/2_ value of ROS was reduced from 2.75 ± 0.70 to 1.99 ± 0.44 and 1.68 ± 0.35 h with downregulation of 28% and 39%. Meanwhile, compared with the control group, the differences in *T*_max_ and MRT were found only in the high-dose GLT group (200 mg/kg).

**Table 4.** Pharmacokinetic parameters of ROS (10 mg/kg) in the control group and the multiple-dose GLT group (*n* = 6).

Parameters	Control	GLT (100 mg/kg)	GLT (200 mg/kg)
*t*_1/2_ (h)	2.75 ± 0.70	1.99 ± 0.44*	1.68 ± 0.35**
CL/F (mL/h)	13.92 ± 3.04	25.54 ± 9.02*	30.00 ± 8.52**
MRT (h)	3.27 ± 0.65	2.95 ± 1.13	2.13 ± 0.28*
AUC_0-t_ (h·μg/mL)	139.69 ± 45.46	84.58 ± 39.87*	66.60 ± 15.90**
*C*_max_ (μg/mL)	38.72 ± 9.73	28.51 ± 5.56*	28.41 ± 4.64*
*T*_max_ (h)	0.79 ± 0.40	0.54 ± 0.25	0.37 ± 0.13*

*p < 0.05, **p < 0.01, compared with control.

### Effects of quercetin, kaempferol, and isorhamnetin on the activity of RHCYP2C8 and RHCYP2C9

**Figure 4.**
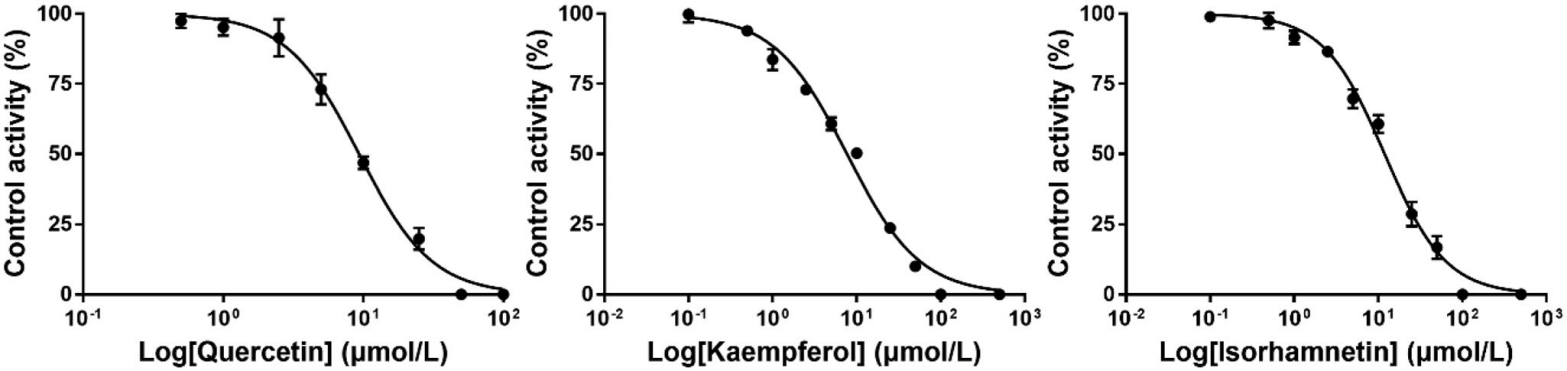
Inhibition curve of CYP2C8 by quercetin, kaempferol, and isorhamnetin (Mean ± SD, *n* = 3).

**Figure 5.**
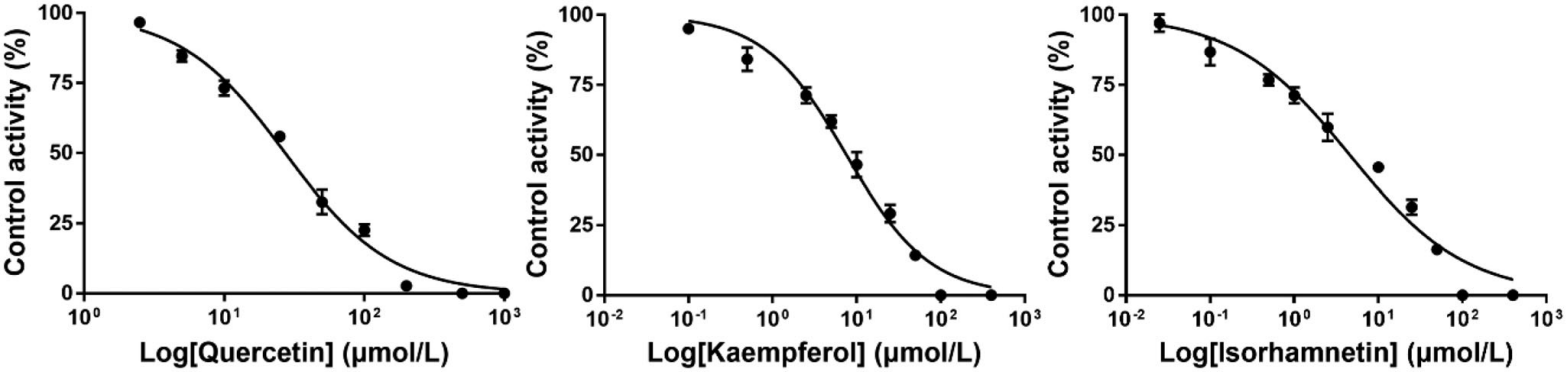
Inhibition curve of CYP2C9 by quercetin, kaempferol, and isorhamnetin (Mean ± SD, *n* = 3).

To elucidate the potential mechanism of the inhibitory effect of a single dose GLT on the pharmacokinetics of ROS in rats, the *in vitro* study with RHCYP2C8 and RHCYP2C9 microsomes was further evaluated. As shown in Figures 4 and 5, we found that kaempferol moderately inhibited CYP2C8 and CYP2C9 activity with respective IC_50_ values of 7.67 and 7.57 μmol/L. Quercetin repressed CYP2C8 activity at a moderate level but regulated CYP2C9 activity towards infirm repression, with IC_50_ values being 9.32 and 27.31 μmol/L, respectively. However, isorhamnetin exerted a weak inhibition on CYP2C8 activity and moderate CYP2C9 inhibition activity, with respective IC_50_ values of 11.90 and 4.59 μmol/L.

### Effects of multiple-dose GLT on CYP2C8 and CYP2C9 activity in rats

**Figure 6.**
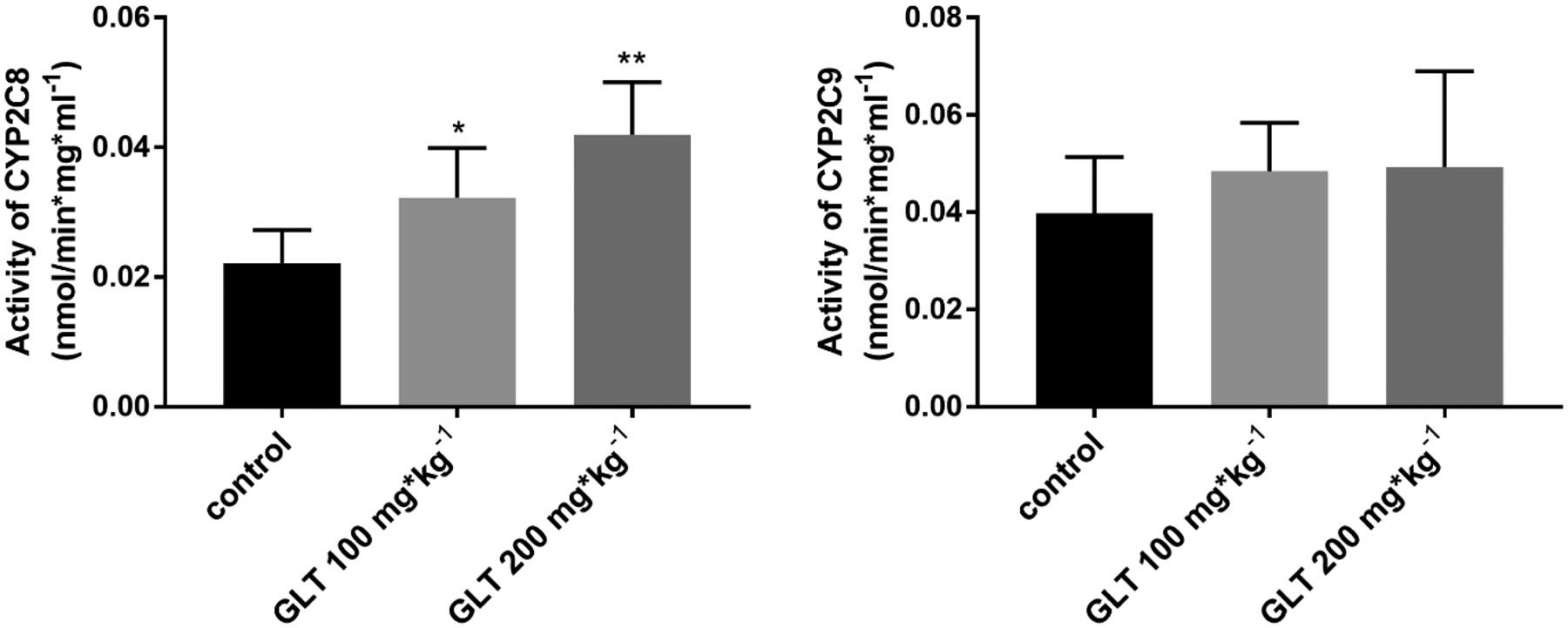
Changes in CYP2C8 and CYP2C9 activities in rats given GLT (100 mg/kg or 200 mg/kg) for 10 days (Mean ± SD, *n* = 5). *p < 0.05, **p < 0.01, compared with control.

GLT pre-treatment at 100 and 200 mg/kg for 10 days significantly increased rat CYP2C8 activity by 44% (*p* < 0.05) and 88% (*p* < 0.01), respectively, but did not affect CYP2C9 activity (*p* > 0.05) (Figure 6).

## Discussion

One of the most critical biological processes impacting the blood concentration of drugs *in vivo* is the intrinsic metabolic clearance mediated by the hepatic CYP450 enzymes (Sathyanarayanan et al. 2020). Drugs are mainly used as substrates, inhibitors, or inducers of CYP450. Enzyme inducers can increase the activity of drug enzymes and reduce the exposure of substrates, resulting in loss of efficacy. Enzyme inhibitors may exceed the therapeutic index and lead to toxicity through downregulation of drug enzymes’ activity and a significant increase in substrate concentration (Di 2017). The FDA guideline from its website has identified and recommended ROS as an appropriate agent to assess the impact of a drug on CYP2C8 activity. ROS is metabolised mainly by CYP2C8 and to a lesser extent by CYP2C9 in the human liver (Kirchheiner et al. 2005). Therefore, drugs which influence CYP2C8 or CYP2C9 enzymes may affect the pharmacokinetics of ROS.

In the present study, we found that compared with the control group, the treatment of ROS with GLT as a single dose inhibited the metabolism of ROS in rats. GLT was found to alter the pharmacokinetic profile of ROS, which could dose-dependently prolong the *t*_1/2_ and MRT of ROS. However, there was no significant difference in AUC_0-t_, *C*_max_, and *T*_max_, which suggested that a single-dose GLT may not influence ROS absorption. To investigate its potential mechanism, the effects of quercetin, kaempferol, and isorhamnetin on the activity of CYP2C8 and CYP2C9 were also investigated using RHCYP2C8 and RHCYP2C9 yeast microsomes. The substance in GLT are ginkgo flavonoids (mainly including quercetin, kaempferol, and isorhamnetin), terpenoids (mainly composed of ginkgolides A and B, and bilobalide), and organic acid (He and Edeki 2004). According to previous research, the flavonoid constituents in GLT are major inhibitors (Gaudineau et al. 2004). The effects of ginkgolides A and B are only pronounced at concentrations greater than 100 μmol/L and therefore the inhibitory effects of terpenoids appear negligible (He and Edeki 2004). The content of organic acid in *Ginkgo biloba* products is too low to achieve the inhibitory effect [EFSA Panel on Additives and Products or Substances used in Animal Feed (FEEDAP) et al. 2021]. Therefore, we chose to test quercetin, kaempferol, and isorhamnetin rather than commercial herbal products, and the IC_50_ of purified components can be expressed in the level of micro-moles per litre and can be compared with known CYP inhibitors. The results showed that the IC_50_ values of quercetin, kaempferol, and isorhamnetin were 9.32, 7.67, and 11.90 μmol/L for RHCYP2C8, respectively. The IC_50_ values of quercetin, kaempferol, and isorhamnetin were 27.31, 7.57, and 4.59 μmol/L for RHCYP2C9, respectively. According to the general rules for intensity classification of CYP enzyme inhibitors, IC_50_ of potent inhibitors is lower than 1 μmol/L, moderate inhibitors are between 1 and 10 μmol/L, and weak inhibitors are over 10 μmol/L (Testa 2007). Our results indicated that quercetin is a moderate inhibitor for CYP2C8 but a weak inhibitor for CYP2C9; kaempferol is a moderate inhibitor for CYP2C8 and CYP2C9; isorhamnetin is a weak inhibitor for CYP2C8 but a moderate inhibitor for CYP2C9. The IC_50_ value of quercetin for CYP2C8 was consistent with the previous finding where the value is 7 μmol/L (Unger and Frank 2004). In general, the three flavonoids in GLT are capable of inhibiting CYP2C9 *in vitro,* which is similar to the previous studies (Gaudineau et al. 2004). Thus, after a single-dose GLT with ROS administration resulted in the inhibition of ROS hepatic metabolism, the occupancy of the main ingredients in GLT binding the active site on the enzymes where ROS should have bound, results in an enhancement of ROS plasma concentration.

To confirm whether multiple doses of GLT could affect the pharmacokinetics of ROS, two different doses of GLT (100 or 200 mg/kg) were given to rats by oral gavage for 10 consecutive days as pre-treatment. The results showed that the pre-treatment with GLT (100 or 200 mg/kg) increased CL/F of ROS by about 80% and 110%, and decreased *t*_1/2_ by 28% and 39% (*p* < 0.05) compared with the control group. Meanwhile, GLT dose-dependently reduced the *C*_max_ and AUC_0-t_ (*p* < 0.05). These results demonstrated that GLT could reduce the absorption of ROS and accelerate the metabolism of ROS in rats. Besides, the activity of CYP2C8 and CYP2C9 was studied by probe substrate assay methods in rat RLM. The results showed that GLT induced CYP2C8 activity in a dose-dependent manner, but not in CYP2C9 activity (Figure 6), which could explain the increase of CL/F and the decrease of the *t*_1/2_ and the concentration of ROS in the multiple-dose study. Umegaki et al. reported that there is a dose-dependently significant induction of the CYP2C9 activity after administration of GLT (Umegaki et al. 2002), and illustrated that GLT pre-treatment reduces the hypoglycaemic effect of tolbutamide. On the contrary, simultaneous treatment of tolbutamide with GLT as a single dose potentiates the hypoglycaemic effect of tolbutamide (Sugiyama et al. 2004). In addition, previous studies have reported that the content and activity of CYP450 are induced markedly by a bilobalide-rich fraction, but not by a flavonoid-rich fraction (Chang et al. 2006; Umegaki et al. 2007; Deng et al. 2008). Therefore, bilobalide may be the major substance in GLT that induces hepatic CYP450. Since there are many substances in GLT, further research will be required to determine which ingredients in GLT are capable of inducing CYP isozymes and explore the potential for the interactions between GLT and other drugs in clinical practice.

## Conclusions

Our study found that GLT alters the pharmacokinetic behaviour of ROS when they are co-administrated. A single-dose GLT administration reduces ROS metabolism by inhibiting CYP2C8 and CYP2C9 activity, while the multiple-dose GLT increases ROS metabolism by inducing CYP2C8 activity in rats. Therefore, it is noteworthy that a single-dose or multiple-dose administration of GLT should be cautious when combined with the drugs metabolised by CYP2C8 or CYP2C9 such as ROS in clinical use.
